# Comparison of clinical practice guidelines for the management of pain, agitation, and delirium in critically ill adult patients

**DOI:** 10.1002/ams2.337

**Published:** 2018-04-10

**Authors:** Ryosuke Tsuruta, Motoki Fujita

**Affiliations:** ^1^ Acute and General Medicine Yamaguchi Graduate School of Medicine Ube Yamaguchi Japan

**Keywords:** Analgesia, ICU, outcome, recommendation, sedation

## Abstract

Guideline‐based management approaches for pain, agitation, and delirium (PAD) in critically ill adult patients are widely believed to result in good outcomes. However, there are some differences in the recommendations and evidence levels among the management guidelines established for PAD. To identify and compare the current management guidelines, we used the PubMed database. The PAD guidelines and Federación Panamericana e Ibérica de Sociedades de Medicina Crítica y Terapia Intensiva (FEPIMCTI) guidelines were identified from our search. We compared the main aspects of these two guidelines as well as the Japanese guidelines for the management of PAD (J‐PAD guidelines). The PAD, FEPIMCTI, and J‐PAD guidelines contained a total of 4, 12, and 5 sections, having 32, 138, and 37 recommendations, respectively, pertaining to routine monitoring of pain in adult patients in the intensive care unit. Intravenous opioids were recommended as the first‐line drug of choice for treating pain. Sedative titrated to maintain a light, rather than deep, level of sedation can be given unless clinically contraindicated. Although neither the PAD nor J‐PAD guidelines recommend use of a pharmacologic delirium prevention protocol or treatment with any pharmacological agent to reduce the duration of delirium, the FEPIMCTI guidelines provide such recommendations. The FEPIMCTI guidelines provide suggestions on which analgesics to use for several different cases and present algorithms for sedation and analgesia. The outlines of the three guidelines are similar, and all reinforce the management of PAD to improve patient outcomes.

## Introduction

The guidelines for sedation and analgesia in critically ill adult patients, published in 2002 by the Society of Critical Care Medicine (USA), have become the best model for subsequent guidelines in individual countries or critical care medicine specialties such as trauma.^1^ These guidelines are remarkable in terms of demonstrating pharmacological and non‐pharmacological strategies for analgesia, sedation, and delirium using the appropriate evaluation scale or tool.^1^ In the case of trauma, the guidelines for sedation and analgesia during mechanical ventilation, published in 2007, recommend prompt and adequate control of pain, agitation, and delirium (PAD), without oversedation, and provide an algorithm for medication management for each component of PAD.^2^ The revised guidelines of the Society of Critical Care Medicine used the title “Management of PAD” and revised the terms “sedation and analgesia” to “pain and agitation”,^3^ and used symptoms and syndromes instead of medical procedures. The guidelines recommend analgesia‐first sedation with a light target level of sedation and evaluation of the pain and agitation/sedation. Analgesia and sedation should not be administered readily without prior evaluation. Furthermore, the guidelines recommend routine monitoring for delirium onset in adult patients in the ICU. The Japanese guidelines for the management of PAD (J‐PAD guidelines) were published after exhaustive consideration of all PAD‐related published guidelines and of Japanese intensive care unit (ICU) conditions (i.e., closed/open ICUs, number of available nurses, and available pharmacological agents).^4^

Hence, we undertook a review and comparison of recently published guidelines for the management of PAD.

## Methods

We used the PubMed Advanced Search Builder (http://www.ncbi.nlm.nih.gov/pubmed/advanced) to identify current guidelines for the management of PAD in critically ill adult patients. The search terms were “guidelines” in the title, “sedation” in the title/abstract, and “adult” in the title/abstract. The publication date was from January 2013 to December 2017. The query was Search (((guidelines [Title]) AND sedation[Title/Abstract]) AND adult[Title/Abstract]) AND (“2013/01”[Date ‐ Publication]: “2017/12”[Date ‐ Publication]). We retrieved 13 articles and finally selected two guidelines.^3^, ^5^ The J‐PAD guidelines were also evaluated alongside these two guidelines for comparison, and the main aspects compared were as follows: basic information about the guidelines (e.g., year of publication and historical background), the guideline development process (e. g., national or international, the authors, and the methods used for evidence evaluation and recommendation development), and recommended strategies.

## Review

### History of the three guidelines

#### Guidelines for PAD

The American College of Critical Care Medicine published a revised version of the PAD guidelines in 2013.^3^ The title “The management of PAD” was changed from the previous title “The sustained use of sedatives and analgesics”^1^ to reinforce a “patient‐centered” approach. The 20‐member multidisciplinary task force of the American College of Critical Care Medicine, including physicians, nurses, and pharmacists, began to revise the 2002 version of these guidelines in 2006. The task force was divided into four subcommittees, pain, sedation, delirium, and related clinical outcomes, and the team leaders of each subcommittee were appointed by the task force chair. The subcommittees were responsible for developing corresponding clinical questions, identifying and evaluating the relevant clinical evidence (December 1999–December 2010), developing guideline statements and recommendations using the GRADE methodology (http://www.gradeworkinggroup.org), and drafting their section of the PAD guidelines.^6^

#### Federación Panamericana e Ibérica de Sociedades de Medicina Crítica y Terapia Intensiva (FEPIMCTI) guidelines

The FEPIMCTI is an association focused on critical care medicine of Spain, Portugal, USA, Mexico, Cuba, Central America, Caribbean nations, Costa Rica, Panama, Colombia, Venezuela, Brazil, Peru, Bolivia, Chile, Paraguay, Argentina, and Uruguay. Twenty‐one specialists (likely all physicians) in critical care medicine from nine countries (Spain, USA, Mexico, Colombia, Venezuela, Brazil, Peru, Chile, and Argentina) were selected by the Society of Critical Care Medicine of each country. The specialists used the 2007 Spanish guidelines as a starting point, because 18 of the members had already contributed to the development of these guidelines.^7^ A search of works published from January 2007 to July 2012 identified more than 1,000 references and was selected by the project coordinator and a methodologist.

#### Japanese PAD guidelines

The Japanese Society of Intensive Care Medicine established a committee to develop the J‐PAD guidelines in 2013. The committee is comprised of 12 members, including five physicians, four nurses, two pharmacists, and one physical therapist. Three physicians had contributed to developing Japanese guidelines for sedation during mechanical ventilation in 2007.^8^ The committee was divided into four groups, pain, sedation, delirium, and rehabilitation therapy and other. Each group member was responsible for developing corresponding clinical questions, identifying and evaluating the relevant clinical evidence (December 1999–February 2013, and January 1996–February 2013 for Japanese published reports), developing guideline statements and recommendations following the PAD guidelines, and drafting their section of the J‐PAD guidelines.

### Comparison of selected topics in the three guidelines

#### Evidence‐based grading system

The composition of the FEPIMCTI guidelines is quite different from those of the others. The FEPIMCTI guidelines are comprised of 12 sections addressing not only the general management of PAD but also the specific management of various patient populations (e.g., patients with kidney failure) (Table 1). The PAD guidelines are comprised of four sections: pain and analgesia, agitation and sedation, delirium, and PAD management strategies to improve ICU outcome. The J‐PAD guidelines have a similar section composition and include the following five sections: pain and analgesia, agitation and sedation, delirium, rehabilitation therapy, and strategies for managing PAD and sleep and performing analgosedation in non‐intubated patients.

**Table 1:** Sections of guidelines published by the Federación Panamericana e Ibérica de Sociedades de Medicina Crítica y Terapia Intensiva for the management of pain, agitation, and delirium in critically ill adult patients

	Title
1	Patients requiring conscious or cooperative sedation
2	Monitorization of sedoanalgesia
3	Patients with delirium and withdrawal syndrome
4	Patients without endotracheal intubation or ventilator support
5	Patients with endotracheal intubation and mechanical ventilation
6	Patients undergoing withdrawal of the endotracheal tube and mechanical ventilation
7	Special populations: trauma patients, elderly subjects, pregnant patients and burn victims
8	Sedoanalgesia in the immediate postoperative period of cardiovascular surgery
9	Neurological and neurocritical patients
10	Patients with kidney or liver failure
11	Patients requiring special procedures (tracheostomy, thoracic catheters or tubes, peritoneal lavage, wound or burn lavage and debridement)
12	Non‐pharmacological strategies or complementary treatments

Both the PAD and J‐PAD guidelines (hereafter referred to as “both PAD guidelines”) involve descriptive and actionable questions and demonstrate the strength of recommendations for actionable questions only (Table 2). There are 32, 138, and 37 recommendations in the PAD, FEPIMCTI, and J‐PAD guidelines, respectively (Table 3). Both PAD guidelines provide 22 answers, with evidence levels, to the descriptive questions. The PAD guidelines have two recommendations of grade 1A for the treatment of neuropathic pain and electroencephalogram monitoring in select neurological patients. The FEPIMCTI guidelines have four recommendations of grade 1A pertaining to sedation and analgesia protocols during ventilator weaning, and to regional and epidural analgesia for certain postoperative and chest trauma patients.

**Table 2:** Grading of recommendations in three clinical guidelines for the management of pain, agitation, and delirium (PAD) in critically ill adult patients

	Description	PAD	FEPIMCTI	J‐PAD
Direction of recommendation	In favor of or for	+	None	+
	Against	−		−
Strength of recommendation	Strong	1	1	1
	Weak	2	2	2
Quality of evidence	High	A	A	A
	Moderate	B	B	B
	Low	C	C	C
Lack of evidence or members’ consensus		0	None	0

Guidelines published by: Society of Critical Care Medicine (USA), 2002, revised 2013 (PAD); Federación Panamericana e Ibérica de Sociedades de Medicina Crítica y Terapia Intensiva (FEPIMCTI), 2013; and Japanese Society of Critical Care Medicine (J‐PAD), 2014.

**Table 3:** Distribution of the recommendations included in three clinical guidelines for the management of pain, agitation, and delirium (PAD) in critically ill adult patients, grouped according to grade

Grade of recommendation	PAD	FEPIMCTI	J‐PAD
1A (%)	2 (6)	4 (3)	0 (0)
1B (%)	9 (28)	60 (44)	12 (32)
1C (%)	3 (9)	50 (36)	5 (14)
2A (%)	0 (0)	0 (0)	0 (0)
2B (%)	5 (16)	10 (7)	4 (11)
2C (%)	6 (19)	14 (10)	7 (19)
0 (%)	7 (22)	0 (0)	9 (24)
Total (%)	32 (100)	138 (100)	37 (100)

Actionable questions only apply to guidelines published by the Society of Critical Care Medicine (USA) (PAD) and the Japanese Society of Critical Care Medicine (J‐PAD). FEPIMCTI, Federación Panamericana e Ibérica de Sociedades de Medicina Crítica y Terapia Intensiva.

#### Pain and analgesia

All critically ill adult patients in medical, surgical, and trauma ICUs typically experience pain, both at rest and under routine ICU care, and have the right to receive adequate pain management where required.^3^, ^5^ Both PAD guidelines recommend (grade +1B) routine monitoring of pain in adult patients in the ICU. Although the FEPIMCTI guidelines recommend using a validated behavioral pain scale to monitor the pain of patients who are unable to communicate, they have yet to recommend (only introduce) using the Behavioral Pain Scale or the Critical‐Care Pain Observation Tool, which are the most valid and reliable pain scales according to both PAD guidelines.^9^, ^10^

Intravenous opioids (fentanyl and morphine in Japan) are recommended as the first‐line drugs of choice for treating pain in ICU patients (grade 1B or +1C). Remifentanil is recommended by the PAD and FEPIMCTI guidelines.

#### Agitation and sedation

The FEPIMCTI and J‐PAD guidelines recommend (grade 1B) prompt identification and treatment of the possible underlying causes of agitation, such as pain, delirium, hypoxemia, hypotension, or withdrawal from alcohol and other drugs in. If the patient requires sedation, sedatives titrated to maintain a light, rather than deep, level of sedation (“conscious or cooperative sedation” in the FEPIMCTI guidelines) can be administered, unless clinically contraindicated. The Richmond Agitation‐Sedation Scale (RASS) and Sedation‐Agitation Scale are the most valid and reliable sedation assessment tools for measuring quality and depth of sedation in adult ICU patients, according to all three guidelines (evidence B).^11^, ^12^ Both PAD guidelines recommend (grade +1B) routine use of either daily sedation interruption or a light target level of sedation for mechanically ventilated adult patients in the ICU.

The use of propofol, dexmedetomidine, or midazolam is recommended. Both PAD guidelines (grade +2B and +2C in PAD and J‐PAD, respectively) prefer sedation using propofol or dexmedetomidine over sedation using midazolam to improve clinical outcomes in mechanically ventilated adult patients. They also recommend (grade +2B) “analgesia‐first sedation” in such patients.

#### Delirium

The FEPIMCTI guidelines recommend (grade 1B) identifying the risk factors for the development of delirium in the ICU and only introduce many risk factors according to previous studies. Whereas the PAD guidelines indicate pre‐existing dementia, history of hypertension and/or alcoholism, and a high severity of illness at admission as risk factors for delirium development, the J‐PAD guidelines indicate pre‐existing dementia, age (elderly), severe infection (sepsis), and a high severity of illness at admission as four baseline risk factors. The reason for the difference is that the Prediction of Delirium in ICU patients (PRE‐DELIRIC) model was not included in the evidence review for the PAD guidelines, because this model was published after December 2010.^13^ However, the PRE‐DELIRIC model was adopted in the FEPIMCTI and J‐PAD guidelines. Both PAD guidelines recommend (grade +1B) routine monitoring of delirium and indicate that the most valid and reliable tools for monitoring delirium in adult ICU patients are the Confusion Assessment Method for the ICU (CAM‐ICU) and the Intensive Care Delirium Screening Checklist (evidence A).^14^, ^15^ In contrast, the FEPIMCTI guidelines prefer the CAM‐ICU over the Intensive Care Delirium Screening Checklist because of the risk of false‐positive cases.

Furthermore, recommendations for the prevention and management of delirium are quite different between the FEPIMCTI guidelines and both PAD guidelines. Both PAD guidelines provide no recommendation for using a pharmacologic delirium prevention protocol or for treatment using any pharmacological agent to reduce the duration of delirium. However, haloperidol, atypical antipsychotics, and dexmedetomidine are recommended (grade 1B) for the management of delirium in the FEPIMCTI guidelines. Early mobilization in non‐pharmacological strategies is recommended (grade 1B) to reduce the incidence and duration of delirium in all three guidelines.

#### Differences between PAD and J‐PAD guidelines

No subtypes of coma (i.e., medication‐related, structural, neurological, and medical) may be a risk factor for the development of delirium in ICU patients according to the J‐PAD guidelines, whereas coma is an independent risk factor according to the PAD guidelines. One study classified delirium into two categories: rapidly reversible, sedation‐related delirium (delirium that abates shortly after sedative interruption) and persistent delirium (delirium that persists despite a short period of sedative interruption).^16^ Rapidly reversible, sedation‐related delirium was found to be associated with fewer ventilator, ICU, and hospital days than was persistent delirium. Patients with persistent delirium had increased 1‐year mortality rates compared with those with no delirium or rapidly reversible, sedation‐related delirium. In another study, the plasma levels of fentanyl and midazolam were higher in comatose patients than in delirious patients.^17^ In contrast, delirious patients had higher levels of the inflammatory mediator interleukin‐6 compared with comatose patients. According to these data, coma appears to be associated with drug exposure, whereas delirium may be associated with systemic inflammation.^17^ Finally, Kress identified the arbitrary method used to distinguish between coma and delirium in the CAM‐ICU tool.^18^ If the RASS score changes from −3 to −4, or if the patient shows a continuous progression (from delirium to coma) with the same pathophysiology as acute brain dysfunction, the patient should be diagnosed as comatose.

#### Differences between PAD and FEPIMCTI guidelines

Deep sedation levels are defined as a RASS score of −3 to −5 in the PAD guidelines versus −4 to −5 in the FEPIMCTI guidelines. Furthermore, the FEPIMCTI guidelines define mild sedation as a RASS score of +1 to −3.

**Figure 1:**
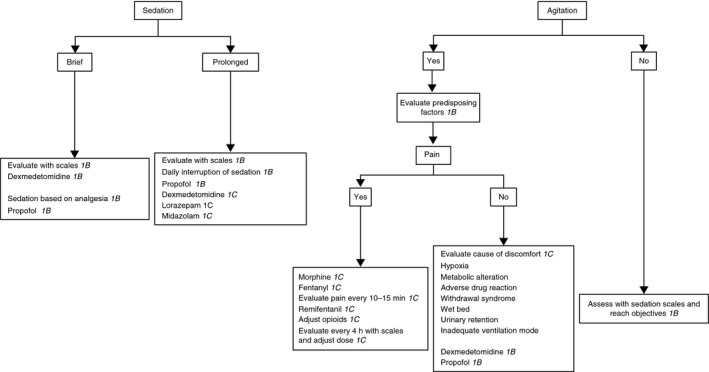
Algorithm for administering sedation and analgesia in patients with tracheal intubation (reproduced from Celis‐Rodríguez *et al*. (2013)^5^ with permission from Elsevier).

The FEPIMCTI guidelines specify which analgesics to use for different patients. Fentanyl is recommended (grade 1C) as the analgesic of choice for hemodynamic instability, bronchial asthma, or chronic obstructive pulmonary disease. Remifentanyl is recommended (grade 1B) for patients weaning from a mechanical ventilator. Ketamine is recommended (grade 1B) as the first‐line medication for sedoanalgesia during routine painful procedures in burn victims. Furthermore, the FEPIMCTI guidelines include an algorithm for sedation and analgesia in patients undergoing tracheal intubation (Fig. 1).

The FEPIMCTI guidelines provide the unique recommendation (grade 1C) of informing the patient about his/her illness and the procedures to be performed. They also advise (grade 1C) establishing an effective means of communication with patients on a mechanical ventilator.

Finally, the FEPIMCTI guidelines address the evaluation and management of analgesia and sedation and risk factors, diagnosis, and management of delirium for all components of PAD together, whereas both PAD guidelines address the assessment, treatment, and prevention of each component of PAD individually.

## Conclusions

The overall composition and recommendations are similar between the PAD and J‐PAD guidelines. Both recommend analgesia‐first sedation, a light target level of sedation, routine monitoring of delirium, and an interdisciplinary ICU approach for managing PAD. The composition of the FEPIMCTI guidelines is quite different from those of both PAD guidelines. The FEPIMCTI guidelines, in 12 sections, provide 138 recommendations as well as algorithms for sedation and analgesia. However, the outline is similar to those of both PAD guidelines, and they also reinforce management of PAD to improve patient outcomes.

## Disclosure

Approval of the research protocol: Not applicable.

Informed consent: Not applicable.

Registry and the registration no. of the study/trial: Not applicable.

Animal studies: Not applicable.

Conflict of interest: None declared.
